# Prevalence of *Chlamydia trachomatis*, *Neisseria gonorrhoeae* and *Trichomonas vaginalis* infection in pregnant adolescent women and its association with pregnancy outcomes

**DOI:** 10.1186/1471-2334-14-S3-E33

**Published:** 2014-05-27

**Authors:** Priti Ghope, Subash C Sonkar, Kirti Wasnik, Pratima Mittal, Daman Saluja

**Affiliations:** 1 Department of Obstetrics & Gynecology Vardhman Mahavir Medical College and Safdarjung Hospital, New Delhi-110029, India; 2 Dr. B. R. Ambedkar Center for Biomedical Research, University of Delhi, Delhi-110007, India

## Background

Sexually transmitted infections (STIs) are major cause for reproductive morbidity, infertility, long term disability and death amongst men, women and infants globally. *T. vaginalis*, *N. gonorrhoeae* and *C. trachomatis* are well established agents of STIs. In present study, we determined the prevalence of these infections in asymptomatic pregnant adolescent women using PCR and studied the association of these infections with maternal and fetal outcome.

## Methods

Dry vaginal swabs from 232 asymptomatic pregnant adolescent women in the age group of 17 to 19 years, attending the obstetrics outpatient clinic of VMMC and Safdarjung Hospital, New Delhi were collected. Genomic DNA was extracted and used as template for PCR amplification using primers targeting *pfoB*, *gyr A and orf1* gene for diagnosis of *T. vaginalis*, *C. trachomatis* and *N. gonorrhoeae*, respectively.

## Results

Out of 232 samples, 14 (6.03 %) women had infection: one woman had mixed infection, 3 women (1.29 %) tested positive for *T. vaginalis*, one women (0.43%) tested positive for *C. trachomatis* and 10 women (4.31 %) were infected with *N. gonorrhoeae* whereas 218 women (93.96%) were uninfected. Amongst 160 patients who have delivered, following clinical outcomes were observed: 44% women gave birth to fetus with low birth weight (2-2.5kg=67(41.35%), 1.5-1.9kg =3(1.85%), <1.5 kg=2 (1.23%), Preterm labour pain=24(15%), Preterm delivery=24 (15%), Leaking pervaginum=12 (7.5%) Premature rupture of membrane=1(.62%), Pneumonia=1(.62%), NICU stay=25(15.62%).

## Conclusion

Guidelines should be formed to screen and treat patients during antenatal care for these infections to avoid adverse maternal and fetal outcomes.

